# Hiatal Hernia With Ulcer at the Gastroesophageal Junction Presenting With Progressive Dysphagia and Epigastric Pain: A Case Report

**DOI:** 10.7759/cureus.63629

**Published:** 2024-07-01

**Authors:** Somya Gupta, Vivek Chakole, Abhiram A Sahasrabhojanee

**Affiliations:** 1 Department of General Medicine, Jawaharlal Nehru Medical College, Datta Meghe Institute of Higher Education and Research, Wardha, IND; 2 Department of Anaesthesiology, Jawaharlal Nehru Medical College, Datta Meghe Institute of Higher Education and Research, Wardha, IND; 3 Department of Medicine, Jawaharlal Nehru Medical College, Datta Meghe Institute of Higher Education and Research, Wardha, IND

**Keywords:** esophagus, hiatal hernia, epigastric pain, acid reflux, dysphagia

## Abstract

A hiatal hernia occurs when the contents of the abdominal cavity, most often the stomach, protrude into the chest cavity through the esophageal hiatus. The hiatus is an elliptical-shaped outlet, typically formed by parts of the right diaphragmatic crus surrounding the distal esophagus. This ailment can transpire due to either the broadening of the specific diaphragmatic opening or a shortening in the overall length of the esophagus, leading to herniation of the stomach into the thoracic region. Raised pressure in the abdominal region may also be one of the culprits. Patients with a hiatal hernia usually remain asymptomatic, but patients might have difficulty swallowing both liquids and solids in the advanced stages of the disease. The disease is rarely accompanied by reflux of gastric acid into the esophagus due to decreased activity of the lower esophageal sphincter, leading to increased complaints of epigastric pain and ulceration near the gastroesophageal junction. Long-standing cases can increase the risk of developing Barrett’s esophagus with dysplasia, which may advance to esophageal carcinoma in later stages. Advanced age and obesity are significant risk factors for hiatal hernia. Obese individuals, in particular, experience higher intra-abdominal pressure, which significantly raises the likelihood of developing a hiatal hernia. The hernia may be diagnosed through an upper gastrointestinal endoscopy or radiologically through a chest X-ray in the posterior-anterior view, defining the border of the esophagus. Hence, this facilitates a more seamless and precise diagnosis. Surgical fundoplication treatment improves the patient’s condition better than solitary medical management. Overall, addressing the condition surgically often yields more favorable outcomes and enhances the patient’s quality of life. Hiatal hernia usually presents with no or minimal clinical manifestations. Thus, this case report highlights the importance of comprehensive clinical management of such cases.

## Introduction

Hiatal hernia is a diaphragmatic defect caused due to raised intra-abdominal pressure causing abdominal organs, especially the stomach, to herniate into the thoracic cavity. It is incidental in about 50% of people over the age of 70 years and quite rare in the fourth and fifth decades of life. Most cases remain asymptomatic throughout their lives and only about 9% of the reported cases present with clinical signs and symptoms [1]. The primary symptom of a hiatal hernia is gastric reflux, exhibited through regurgitation and heartburn. Less typical manifestations include epigastric pain and chronic anemia due to the malabsorption of iron [2]. A larger hernia bigger than 2 cm may showcase dysphagia and early onset of regurgitation. Reflux symptoms may present with continuous exposure of the esophagus to gastric acid leading to esophagitis [3]. Type I hiatal hernias, also termed sliding hernias, are the most prevalent and primarily significant due to their association with reflux diseases. The less common types II, III, and IV hiatal hernias are classified as para-esophagal hernias. Collectively, these types account for only 5-15% of all hiatal hernias [4]. Diagnosis is exhibited through radiography performed via barium swallow, which gives practical data about the extent of the stomach relocated into the esophagus and the exact site of the gastroesophageal junction. Although barium swallow is an invasive technique, it remains an essential diagnostic modality for hiatal hernia [5]. Another important modality includes endoscopy of the esophagus and stomach, which shows the present condition of the esophageal mucosa [6].

## Case presentation

A 54-year-old woman presented to the outpatient department of a tertiary health care center with chief complaints of difficulty swallowing food for one month. The patient also complained of pain in the epigastrium region for 20 days. The patient was alright one month back when she started to experience difficulty swallowing. The dysphagia was insidious in onset and gradually progressive. Initially, it was intermittent and mild but had worsened over the past month and progressed to occurring with both solids and liquids. The pain in the epigastrium was described as a burning sensation exacerbated by meals and lying down straight. It was relieved by sitting upright and using antacids. The patient had a past medical history of gastroesophageal reflux disease, for which the patient was prescribed proton pump inhibitor therapy.

There was no history of hematemesis, melena, unintentional weight loss, or anorexia. The patient denied any history of chest pain, orthopnea, or paroxysmal nocturnal dyspnoea, ruling out any cardiac pathology. There was no history of any prior gastrointestinal surgeries. Additionally, the patient did not report any known food allergies or intolerance and denied the use of non-steroidal anti-inflammatory drugs and alcohol. The patient’s family history was not significant. The patient had no history of hypertension, diabetes mellitus, tuberculosis, or asthma. Moreover, there was no history of any blood transfusion, trauma, or recent hospitalization.

Clinical examination

As the patient arrived at the outpatient department with the complaint of dysphagia and abdominal pain, physical examinations and investigations were conducted for the precise diagnosis of the case. The patient was conscious, cooperative, and well-oriented regarding time, place, and person. The patient seemed uncomfortable and fatigued. She showed no signs of cyanosis, pallor, icterus, lymphadenopathy, clubbing, or edema. The patient demonstrated a blood pressure of 108/75 mmHg, heart rate of 63 beats per minute, temperature of 97.5°F, and respiratory rate of 16 cycles per minute.

A local examination of the abdomen was conducted to learn more about the underlying cause of dysphagia and abdominal pain. Table 1 exhibits the findings of the systemic examination of the abdomen conducted in detail.

**Table 1:** Clinical findings of local examination of the abdomen.

Examinations	Examination findings of the abdomen
Inspection	The abdomen was boat-shaped, which is the normal shape of the abdomen. The umbilicus was present centrally in the mid-abdomen and inverted. The distance between the xiphisternum and pubic symphysis was equal. Abdominal movements were normal, as the abdomen bulged during inspiration and falls during expiration. No visible pulsations and no dilated veins on the abdomen were present. The skin over the abdomen showed no signs of any hyper or hypopigmentation present over the skin. There were no stretch marks or surgical scars over the abdomen
Palpation	The abdomen was soft to the touch with no signs of masses, swelling, tenderness, or rigidity. The liver presented normally with sharp, firm, and regular edges. The surface seemed smooth. The spleen was not palpable and normal in presentation The gallbladder was not palpable and normal in presentation The kidney was not palpable and normal in presentation.
Percussion	There were no signs of shifting dullness, horseshoe dullness, fluid thrill, and puddle sign
Auscultation	Normoactive sounds of the bowel were heard in all four quadrants. The bowel sounds were low-pitched and gurgling in nature. The rate was three sounds per minute

Diagnostic modalities

Several diagnostic methods are available for detecting hiatal hernias. Definitive diagnosis can be made through endoscopic and radiographic findings. These findings have a selective criterion that helps rule out other diseases. Diagnosing a hiatal hernia can be tedious as the location of the esophageal junction may shift during normal physiological events such as deglutination, breathing, and changes in the patient’s position.

Endoscopic Findings

**Figure 1:**
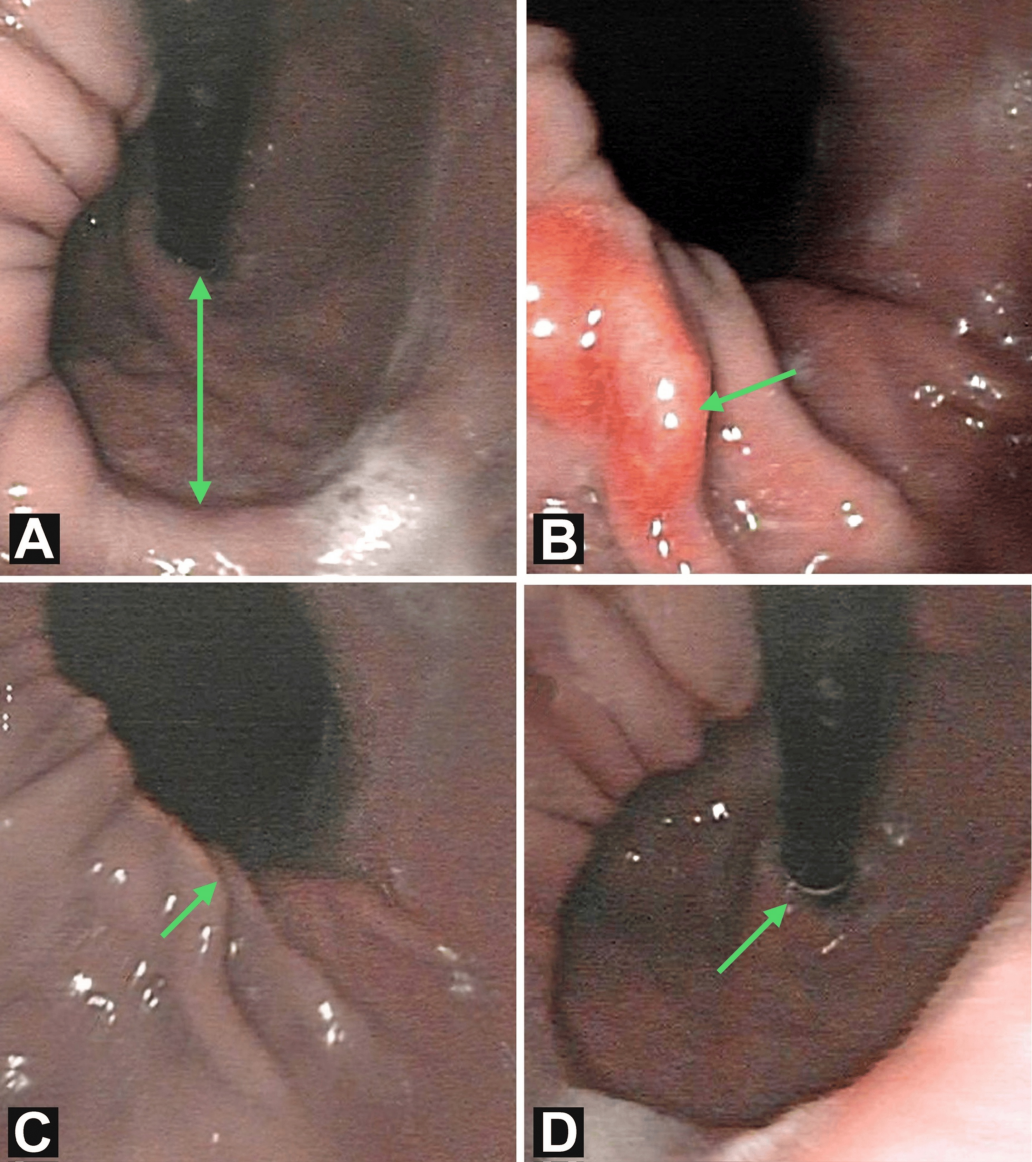
Retrograde view of flexible endoscope suggestive of (A) distance of more than 2 cm between the gastroesophageal junction (GEJ) and diaphragmatic hiatus; (B) signs of ulceration near the GEJ; (C) diaphragmatic hiatus; (D) endoscope entering through the GEJ into the cardia region of the stomach.

Endoscopic impressions were highly diagnostic and presented several findings about the present condition of the upper gastrointestinal tract. A flexible endoscope was used to visualize the upper gastrointestinal tract, especially the esophagus. Along with the contour of the esophagus, this method also demonstrated the present condition of the mucosal lining of the gastrointestinal tract. Figure 1 indicates the presence of a large type I hiatal hernia. In addition to the hiatal hernia, there were signs of ulceration at the gastroesophageal junction. Signs of mild congestion were also seen in the antral part of the stomach which suggested frequent episodes of gastric reflux. The distance between the diaphragmatic hiatus and the gastroesophageal junction was greater than 2 cm, confirming the diagnosis.

Endoscopic findings of the gastrointestinal tract conducted are presented in Table 2. These findings were conclusive of a large hiatal hernia with congestion in the antral region along with signs of ulceration at the gastroesophageal junction.

**Table 2:** Endoscopic findings of the upper gastrointestinal tract.

Upper gastrointestinal endoscopy site	Findings
Esophagus	Z line was present at 40 cm from the start. Hiatal hernia was present. Ulceration was noted at the gastroesophageal junction. Reflux esophagitis and oesophageal varices were absent
Stomach	The antral mucosa of the stomach was congested. The fundal and body of the stomach mucosa were normal. The cardiac and pylori channels were also normal
Duodenum	Mucosa of the first and the second part of the duodenum was normal
Larynx	The vocal cords were white and normal with usual movements. Valleculae was normal. Pyriform fossae were clear and had no growth up to the vocal cord along with a normal cricopharyngeal opening

Radiological Findings

**Figure 2:**
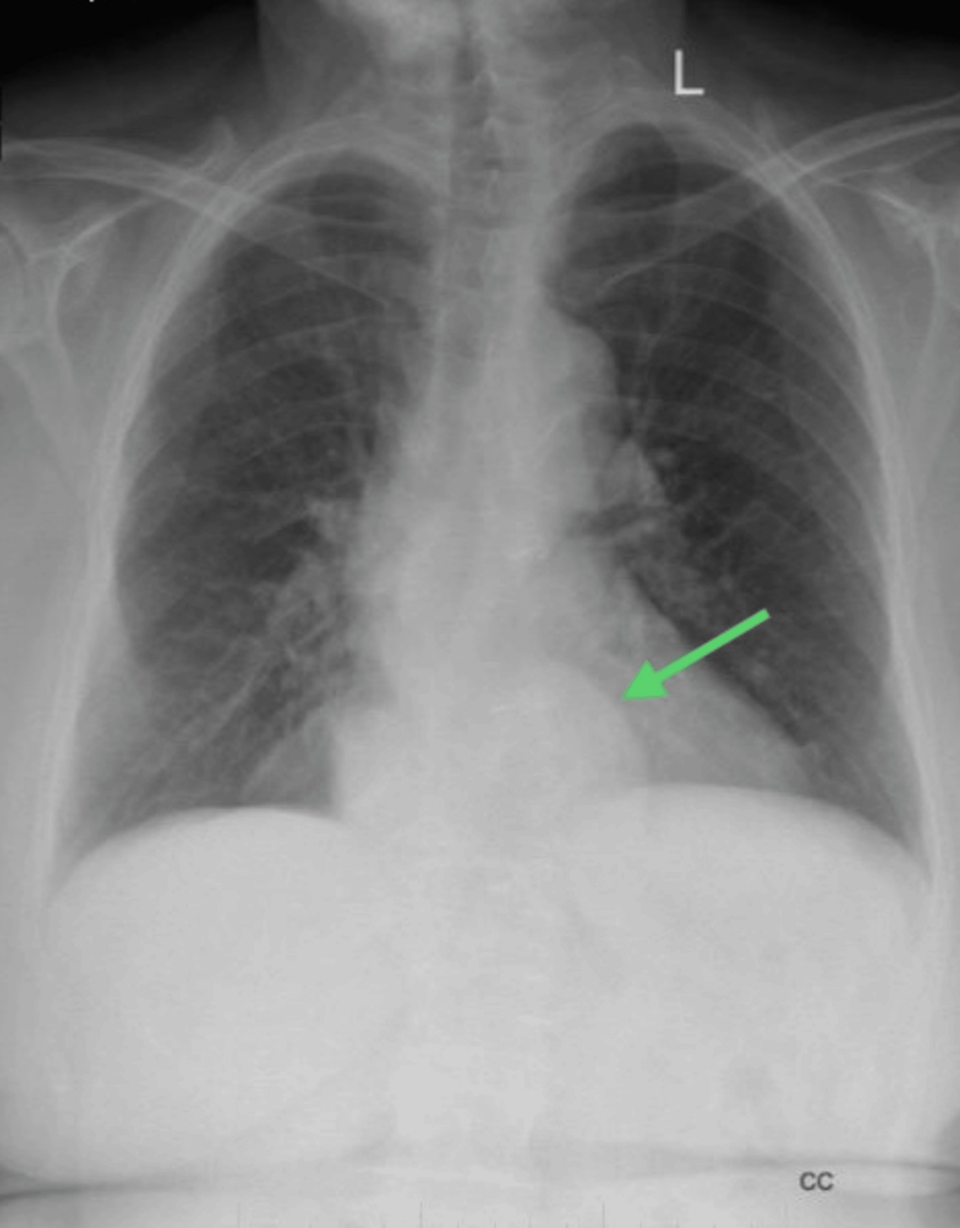
X-ray of the thoracic cavity in posterior-anterior view. The green arrow represents a retrocardiac mass with no air-fluid levels, which is highly suggestive of a hiatal hernia.

Radiological imaging included a chest X-ray conducted in the posterior-anterior view. A hiatal hernia was recognized when the separation between the gastroesophageal junction and the diaphragm hiatus was 2 cm or more. Figure 2 suggests well-defined opacity in a retrocardiac location with indistinct medial and anteromedial borders. Chest X-ray showed mediastinal enlargement due to the hiatal hernia. The hiatus was positioned on the patient’s left side and no air-fluid levels could be noted in the X-ray findings. The visualized lung fields were normal with clear costophrenic angles. The positioning of the trachea and main bronchi seemed normal, with no congestion. Finally, the visualized bones and soft tissues also seemed normal.

Management

The management of a case of hiatal hernia includes a multidisciplinary approach with a combination of medical management, lifestyle modification, and potentially surgical management. The final approach of the treatment depends on several varying factors which include the severity of symptoms, size, and type of hernia along with the presence of complications which include perforation, bleeding, obstruction, or strangulation.

Lifestyle Modifications

Lifestyle changes were advised to the patient for a better and faster recovery. Reduction of weight in obese patients is a requirement as it helps in lowering intrathoracic pressure. The patient was advised to maintain a healthy diet rich in good cholesterol, fiber, and proteins. The patient was suggested to take small and frequent meals instead of large and periodic meals. Smoking cigarettes and consumption of alcohol were advised to be stopped immediately as they may cause irritation and worsen the symptoms.

Medical Management

Medical management plays a pivotal role in the management of hiatal hernia. The patient was prescribed antacids and prokinetics to provide symptomatic relief. The primary goal was to reduce gastric acid secretion. A dose of 40 mg of pantoprazole (a proton pump inhibitor) and a dose of 1.5 g of cimetidine (a histamine type 2 receptor blocker) were given to the patient. Additionally, sucralfate in the form of syrup was prescribed to create a protective barrier over the ulcer.

Surgical Management

Surgical repair is the only definitive management for hiatal hernia with gastroesophageal junction ulcer. Numerous surgical procedures can be applied for the management and restoration of the hernia. The patient was suggested to undergo a hiatal repair surgery followed by fundoplication, which is considered the most successful form of treatment.

Surgery Notes

The patient was first brought into the preoperative room after obtaining written consent, along with clearance by the appointed anesthetist. After inducing the patient with general anesthesia and proper positioning, the required number of small incisions were made in the abdominal wall to allow the insertion of the laparoscopic instruments. After locating the hiatus and its extent, the transpositioned stomach was carefully reduced back into the abdominal cavity. The diaphragmatic hiatus was then approximated with sutures to reduce the size of the defect, thus preventing the recurrence of the hernia. Subsequently, a Nissen fundoplication was performed, which involved wrapping a part of the gastric fundus around the lower part of the esophagus to reinforce the lower esophageal sphincter and prevent gastroesophageal reflux. It is a procedure that involves 360-degree wrapping around the esophagus. Postoperative care was provided and the patient was assessed for any bleeding sites. The incisions were closed with absorbable sutures, and sterile dressings were applied. The overall procedure was successful, with no acute complications. The patient was transferred to her room after ensuring stable vitals and was advised for a proper follow-up.

## Discussion

Hiatal hernias involve abdominal contents mainly stomach herniating through the esophageal hiatus. They are mainly classified into three types, namely, sliding type, rolling type, and mixed type, each with characteristic features and symptoms [7]. Hiatal hernia impacts the lower esophageal sphincter by shortening the portion exposed to intra-abdominal pressure, reducing the pressure of the lower esophageal sphincter, and impairing the diaphragmatic sphincter. This increases the risk of gastroesophageal reflux and complications. Additionally, large hiatal hernias reduce the peristaltic amplitude and degrade the acid clearance mechanism of the stomach [8]. The larger size of the hiatal hernia presents significant clinical manifestations, as the majority of patients with severe esophagitis have a history of large hiatal hernia [9]. As gastric reflux diseases often manifest in patients with hiatal hernia, lifestyle improvements such as weight loss are essential. Initial remedies for symptomatic patients usually comprise antacids, prokinetics, and H2-receptor antagonists, with the latter being vital for effective acid suppression and forming the cornerstone of the treatment. Hence, providing patients with symptomatic relief [10]. However, this deformity may cause substantial morbidity and may not respond to traditional medical treatment. The optimal choice of treatment remains a surgical approach toward the abdomen involving resection of the hernia [11].

## Conclusions

This case study focuses on the patient’s journey through the various stages of hiatal hernia, initially manifesting with reflux-like symptoms that eventually progress to dysphagia and gastritis in long-standing cases. Though it often presents as asymptomatic in the early stages with minor complaints of heartburn, it may progress to esophageal and extra-esophageal complications in the later stages. Hence, timely intervention is required in hiatal hernia cases. This study highlights the importance of various diagnostic modalities available to the patient to achieve a precise diagnosis. We also highlight the spectrum of treatment alternatives available to the patient, from invasive to non-invasive methods. Yet, surgical intervention is the most definitive and associated with the lowest recurrence rates. The necessity of early identification and prompt treatment is paramount to mitigate morbidity associated with hiatal hernias. Therefore, establishing a comprehensive treatment and follow-up program is crucial for the patient’s health and long-term outcomes. Moreover, this study advocates for ongoing research into more minimally invasive, diagnostic, and therapeutic modalities to improve patient care and outcomes in the foreseeable future.
